# The Sufferings of Christ

**Published:** 1887-10-22

**Authors:** 


### WORDS OF CONSOLATION.

[Communications for this column should be addressed, "Chaplain," care of the Editor of THE HOSPITAL, who will gratefully welcome any suggestions or inquiries from matrons, nurses, and the staff generally.]

### LV.—THE SUFFERINGS OF CHRIST.

(*For Reading to the Sick.*)

JESUS held His peace (MATT. xxvi, 63). We must not think that we follow our Blessed Lord very closely, or that we can have much fellowship with Him in His sufferings, until we have learnt the practice of silence on certain occasions when most people insist upon using their tongues. To stand being spoken against, and not to return the charge, is a mark of Christ's disciple. If it has been your way hitherto, or (as you may have supposed) your right, your strength, to retaliate when unkind things are said; or if you have felt how you must have the last word in an argument, for fear of being humbled, or because your opinion seems so worthy of credit; then try next time, under such circumstances, to be on the side of the meek, silent, yet majestic Sufferer. Your opponents may wonder. The world very often misunderstands meekness altogether, and thinks that those who do not insist upon giving back word for word, are wanting in courage, or afraid to speak up. Be it so. Nevertheless our Saviour was not silent under such grievous insults, in order that we might take unlimited revenge with our tongues and be forgiven. He was silent that we might have an example, as well as benefit by an Atonement. In following His example, we must not shut our eyes to the fact that He held His peace at the very moments when most people would have considered themselves thoroughly justified in letting their tongues loose. The very occasions when we think we are bound to defend ourselves are sometimes really the opportunities for a victory through silence. You know how in the case of an intemperate person, if there is to be any permament reform, it can only be accomplished by having a strict rule as to a limited quantity of alcohol, while many authorities believe total abstinence to be the only safeguard. Is it not wonderful that many people who suffer grievously for their intemperance in the matter of language, and inflict at the same time such terrible injuries upon their fellow-creatures, should not see that some rule about abstaining from conversation, under certain circumstances, is absolutely necessary? It may be harder to make a rule not to speak than not to drink, at certain times; yet nothing is too hard with the example of Christ before us, if His Grace is really sought in the imitation of the details of His Passion. Yes, it is hard, this bringing the science of the Cross down to practice. But, remember, if you talk whenever you feel inclined, or whenever you are provoked, or whenever the Tempter whispers that you have a right to speak, you will never be very like Jesus Christ. If you ever seem to be the loser by holding your peace, it may be a deep consolation to reflect that "silence is better than sin."

(*To be continued.*)

